# (*E*)-*N*′-[4-(Di­methyl­amino)­benzyl­idene]-2-(4-methyl­phen­oxy)acetohydrazide

**DOI:** 10.1107/S1600536813034879

**Published:** 2014-01-15

**Authors:** M. K. Usha, S. Madan Kumar, Nitinchandra, B. Kalluraya, N. K. Lokanath, D. Revannasiddaiah

**Affiliations:** a Department of Studies in Physics, University of Mysore, Manasagangotri, Mysore 570 006, India; b Department of Studies in Chemistry, Mangalore University, Mangalagangotri, Mangalore 574 199, India

## Abstract

In the title compound, C_18_H_21_N_3_O_2_, the dihedral angle between the benzene rings is 68.85 (11)°. In the crystal, the mol­ecules are linked by C—H⋯O and N—H⋯O hydrogen bonds, as well as weak C—H⋯π contacts, forming a three-dimensional supra­molecular architecture.

## Related literature   

For biological background to hydrazone derivatives, see: Nithinchandra *et al.* (2012 ▶, 2013 ▶); Holla *et al.* (1992 ▶); Kalluraya *et al.* (1995 ▶). For related structures, see: Sarfraz *et al.* (2010 ▶); Fun *et al.* (2011 ▶).
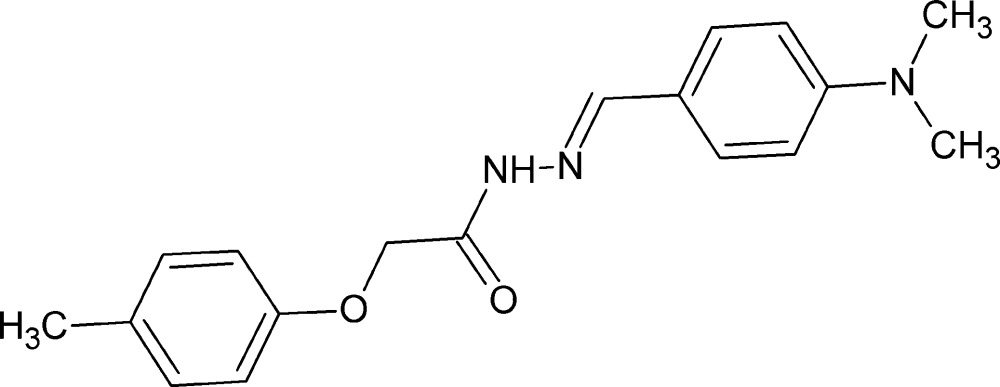


## Experimental   

### 

#### Crystal data  

C_18_H_21_N_3_O_2_
*M* *_r_* = 311.38
Monoclinic, 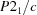

*a* = 11.2237 (6) Å
*b* = 9.4471 (5) Å
*c* = 15.8785 (9) Å
β = 100.868 (3)°
*V* = 1653.42 (16) Å^3^
*Z* = 4
Cu *K*α radiation
μ = 0.67 mm^−1^
*T* = 296 K
0.23 × 0.22 × 0.21 mm

#### Data collection  

Bruker X8 Proteum diffractometer
Absorption correction: multi-scan *SADABS* (Bruker, 2013 ▶) *T*_min_ = 0.862, *T*_max_ = 0.873
13214 measured reflections
2725 independent reflections
2318 reflections with *I* > 2σ(*I*)
*R*_int_ = 0.069

#### Refinement  

*R*[*F* ^2^ > 2σ(*F* ^2^)] = 0.075
*wR*(*F* ^2^) = 0.211
*S* = 1.05
2725 reflections
211 parameters
H-atom parameters constrained
Δρ_max_ = 0.43 e Å^−3^
Δρ_min_ = −0.43 e Å^−3^

### 

Data collection: *APEX2* (Bruker, 2013 ▶); cell refinement: *SAINT* (Bruker, 2013 ▶); data reduction: *SAINT*; program(s) used to solve structure: *SHELXS97* (Sheldrick, 2008 ▶); program(s) used to refine structure: *SHELXL97* (Sheldrick, 2008 ▶); molecular graphics: *Mercury* (Macrae *et al.*, 2008 ▶); software used to prepare material for publication: *PLATON* (Spek, 2009 ▶).

## Supplementary Material

Crystal structure: contains datablock(s) global, I. DOI: 10.1107/S1600536813034879/xu5759sup1.cif

Structure factors: contains datablock(s) I. DOI: 10.1107/S1600536813034879/xu5759Isup2.hkl

Supporting information file. DOI: 10.1107/S1600536813034879/xu5759Isup3.cml

CCDC reference:

Additional supporting information:  crystallographic information; 3D view; checkCIF report

## Figures and Tables

**Table 1:** Hydrogen-bond geometry (Å, °) *Cg*1 and *Cg*2 are the centroids of the C2–C7 and C11–C16 rings, respectively.

*D*—H⋯*A*	*D*—H	H⋯*A*	*D*⋯*A*	*D*—H⋯*A*
N1—H1⋯O2^i^	0.86	2.12	2.952 (2)	163
C8—H8*B*⋯O2^i^	0.97	2.43	3.303 (2)	149
C8—H8*A*⋯*Cg*2^ii^	0.97	2.65	3.442 (2)	139
C16—H16⋯*Cg*1^i^	0.93	2.71	3.394 (2)	131

Symmetry codes: (i) 
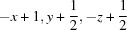
; (ii) 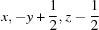.

**Fig. 1.**
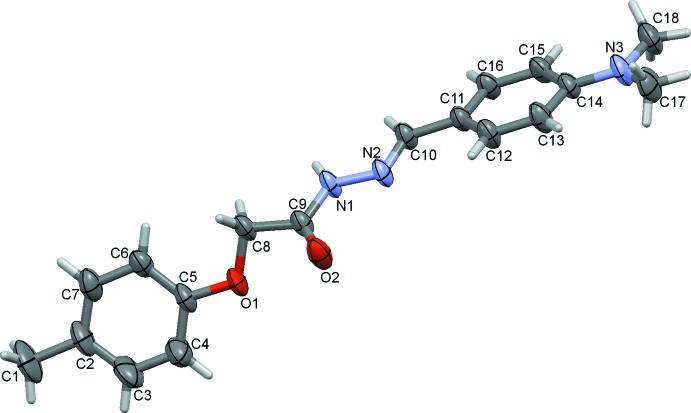
The molecular structure of the title compound with 50% probability ellipsoids.

**Fig. 2.**
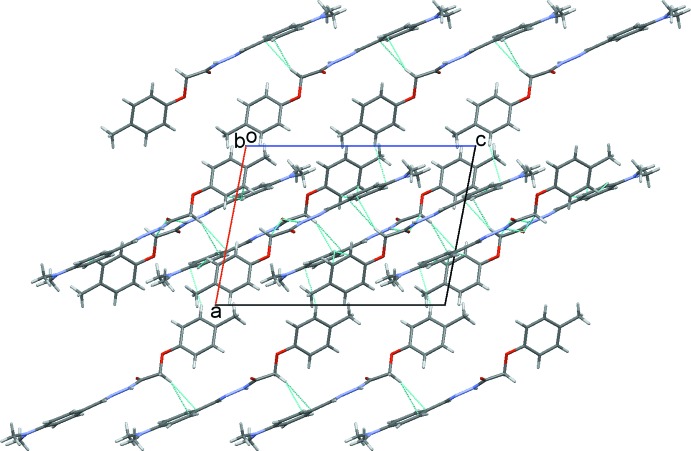
Packing diagram of molecule, viewed along b axis.

## supplementary crystallographic information

### 1. Comment

Hydrazone derivatives possessing an azomethine –NHN=CH– moiety constitute an important class of compounds for new drug development (Nithinchandra *et al.*, 2012). A large number of hydrazone derivatives have been reported to have bactericidal (Holla *et al.*, 1992), fungicidal (Kalluraya *et al.*, 1995) and anticancer (Nithinchandra *et al.*, 2013) activities. As part of our studies in this area, herewith we report the structure of the title compound.

The *ORTEP* of the title compound is shown (Fig. 1) and the dihedral angle between the two phenyl rings is 68.85 (11)°. The overall geometry of the title compound are similar to related structures *N*-{(*E*)-[4-Dimethylamino)phenyl]-methylidene}-2,3-dimethylaniline (Sarfraz *et al.*, 2010) and 2-(4-Methylphenoxy)acetohydrazide (Fun *et al.*, 2011). In the crystal structure, the molecules are connected with intermolecular hydrogen bonds C8—H8B···O2 and N1—H1···O2. They form infinite chains along *b*-axis (Fig. 2 and Table. 1). In addition, short contacts C8—H(8 A)···*Cg*(2) with distance 3.442 (2) Å (angle 139°) [*x*, *y* - 1/2, *z* + 1/2] and C16—H16···*Cg*1 with distance 3.394 (2) Å (angle 131°) [*x* - 1, *y* + 1/2, *z* - 1/2] are observed where *Cg*(1): C2—C7 and *Cg*(2): C11—C16.

### 2. Experimental

To a solution of 2-(4-methylphenoxy)acethydrazide (0.01 mol) in a mixture of DMF and ethanol (10 ml), 4-(dimethylamino)benzaldehyde (0.01 mol) was added. Concentrated sulfuric acid (0.5 ml) was added to this reaction mixture. The contents were refluxed for about 1 h. The solid product separated was collected by filtration. It was dried and recrystallized from ethanol. Single crystals suitable for X-ray analysis were obtained by slow evaporation of an ethanol-*N*,*N*-dimethylformamide (DMF) (3:1) solution.

### 3. Refinement

H atoms were placed in calculated positions with C–H = 0.93–0.97 Å and N–H = 0.86 Å, and refined as riding on their parent C and N atoms with *U*_iso_(H) = 1.5*U*_eq_(C) for methyl H atoms and 1.2*U*_eq_(C,*N*) for the others.

### Crystal data

**Table:**

C_18_H_21_N_3_O_2_	*F*(000) = 664
*M**_r_* = 311.38	*D*_x_ = 1.251 Mg m^−^^3^
Monoclinic, *P*2_1_/*c*	Cu *K*α radiation, λ = 1.54178 Å
Hall symbol: -P 2ybc	Cell parameters from 2725 reflections
*a* = 11.2237 (6) Å	θ = 4.0–64.7°
*b* = 9.4471 (5) Å	µ = 0.67 mm^−^^1^
*c* = 15.8785 (9) Å	*T* = 296 K
β = 100.868 (3)°	Block, red
*V* = 1653.42 (16) Å^3^	0.23 × 0.22 × 0.21 mm
*Z* = 4	

### Data collection

**Table:**

Bruker X8 Proteum diffractometer	2725 independent reflections
Radiation source: Bruker MicroStar microfocus rotating anode	2318 reflections with *I* > 2σ(*I*)
Helios multilayer optics monochromator	*R*_int_ = 0.069
Detector resolution: 10.7 pixels mm^-1^	θ_max_ = 64.7°, θ_min_ = 4.0°
φ and ω scans	*h* = −12→13
Absorption correction: multi-scan *SADABS* (Bruker, 2013)	*k* = −11→5
*T*_min_ = 0.862, *T*_max_ = 0.873	*l* = −18→18
13214 measured reflections	

### Refinement

**Table:**

Refinement on *F*^2^	Secondary atom site location: difference Fourier map
Least-squares matrix: full	Hydrogen site location: inferred from neighbouring sites
*R*[*F*^2^ > 2σ(*F*^2^)] = 0.075	H-atom parameters constrained
*wR*(*F*^2^) = 0.211	*w* = 1/[σ^2^(*F*_o_^2^) + (0.1529*P*)^2^ + 0.2569*P*] where *P* = (*F*_o_^2^ + 2*F*_c_^2^)/3
*S* = 1.05	(Δ/σ)_max_ = 0.002
2725 reflections	Δρ_max_ = 0.43 e Å^−^^3^
211 parameters	Δρ_min_ = −0.43 e Å^−^^3^
0 restraints	Extinction correction: *SHELXL*, FC^*^=KFC[1+0.001XFC^2^Λ^3^/SIN(2Θ)]^-1/4^
Primary atom site location: structure-invariant direct methods	Extinction coefficient: 0.0102 (18)

### Special details

**Table:**

Geometry. Bond distances, angles *etc*. have been calculated using the rounded fractional coordinates. All su's are estimated from the variances of the (full) variance-covariance matrix. The cell e.s.d.'s are taken into account in the estimation of distances, angles and torsion angles
Refinement. Refinement on *F*^2^ for ALL reflections except those flagged by the user for potential systematic errors. Weighted *R*-factors *wR* and all goodnesses of fit *S* are based on *F*^2^, conventional *R*-factors *R* are based on *F*, with *F* set to zero for negative *F*^2^. The observed criterion of *F*^2^ > σ(*F*^2^) is used only for calculating -*R*-factor-obs *etc*. and is not relevant to the choice of reflections for refinement. *R*-factors based on *F*^2^ are statistically about twice as large as those based on *F*, and *R*-factors based on ALL data will be even larger.

### Fractional atomic coordinates and isotropic or equivalent isotropic displacement parameters (Å^2^)

**Table:**

	*x*	*y*	*z*	*U*_iso_*/*U*_eq_	
O1	0.69278 (13)	0.22073 (19)	0.19031 (9)	0.0526 (6)	
O2	0.53999 (16)	0.07569 (16)	0.28054 (9)	0.0536 (6)	
N1	0.47999 (15)	0.29874 (18)	0.30708 (10)	0.0404 (5)	
N2	0.43122 (16)	0.25856 (19)	0.37754 (10)	0.0411 (6)	
N3	0.19247 (18)	0.2905 (2)	0.71623 (11)	0.0513 (7)	
C1	0.9297 (3)	0.2897 (4)	−0.0930 (2)	0.0937 (14)	
C2	0.8634 (2)	0.2746 (3)	−0.01891 (16)	0.0601 (9)	
C3	0.9184 (2)	0.2062 (4)	0.05636 (18)	0.0714 (10)	
C4	0.8604 (2)	0.1913 (3)	0.12481 (16)	0.0622 (9)	
C5	0.74458 (19)	0.2438 (2)	0.11927 (12)	0.0438 (7)	
C6	0.6868 (2)	0.3118 (2)	0.04553 (13)	0.0453 (7)	
C7	0.7476 (2)	0.3253 (2)	−0.02276 (15)	0.0527 (8)	
C8	0.56985 (18)	0.2615 (2)	0.18410 (12)	0.0414 (6)	
C9	0.52905 (19)	0.2017 (2)	0.26216 (12)	0.0392 (6)	
C10	0.39327 (18)	0.3609 (2)	0.41754 (12)	0.0399 (6)	
C11	0.33822 (18)	0.3394 (2)	0.49233 (12)	0.0384 (6)	
C12	0.3056 (2)	0.2065 (2)	0.51882 (14)	0.0456 (7)	
C13	0.2569 (2)	0.1908 (2)	0.59149 (14)	0.0474 (7)	
C14	0.23959 (18)	0.3070 (2)	0.64313 (13)	0.0409 (6)	
C15	0.2715 (2)	0.4407 (2)	0.61621 (13)	0.0434 (6)	
C16	0.31855 (19)	0.4555 (2)	0.54258 (13)	0.0428 (7)	
C17	0.1550 (3)	0.1517 (3)	0.74088 (16)	0.0636 (9)	
C18	0.1782 (2)	0.4102 (3)	0.76985 (15)	0.0612 (9)	
H1	0.47880	0.38630	0.29210	0.0490*	
H1A	1.00830	0.24650	−0.07820	0.1410*	
H1B	0.93890	0.38830	−0.10510	0.1410*	
H1C	0.88410	0.24400	−0.14280	0.1410*	
H3	0.99620	0.16970	0.06030	0.0860*	
H4	0.89910	0.14610	0.17450	0.0740*	
H6	0.60880	0.34760	0.04170	0.0540*	
H7	0.70870	0.37010	−0.07260	0.0630*	
H8A	0.52070	0.22370	0.13210	0.0500*	
H8B	0.56260	0.36380	0.18310	0.0500*	
H10	0.40110	0.45270	0.39820	0.0480*	
H12	0.31690	0.12710	0.48670	0.0550*	
H13	0.23490	0.10090	0.60690	0.0570*	
H15	0.26080	0.52040	0.64840	0.0520*	
H16	0.33790	0.54560	0.52580	0.0510*	
H17A	0.08590	0.12040	0.70000	0.0950*	
H17B	0.13400	0.15720	0.79670	0.0950*	
H17C	0.22040	0.08570	0.74230	0.0950*	
H18A	0.25520	0.45570	0.78800	0.0920*	
H18B	0.14790	0.37840	0.81920	0.0920*	
H18C	0.12190	0.47610	0.73800	0.0920*	

### Atomic displacement parameters (Å^2^)

**Table:**

	*U*^11^	*U*^22^	*U*^33^	*U*^12^	*U*^13^	*U*^23^
O1	0.0494 (9)	0.0749 (12)	0.0419 (8)	0.0043 (8)	0.0300 (7)	0.0077 (7)
O2	0.0804 (11)	0.0332 (9)	0.0576 (9)	0.0002 (7)	0.0400 (8)	−0.0008 (6)
N1	0.0560 (10)	0.0323 (9)	0.0426 (9)	−0.0035 (7)	0.0339 (8)	−0.0003 (7)
N2	0.0524 (10)	0.0392 (10)	0.0400 (9)	−0.0055 (8)	0.0303 (8)	0.0012 (7)
N3	0.0648 (12)	0.0524 (12)	0.0479 (10)	−0.0071 (9)	0.0394 (9)	−0.0023 (8)
C1	0.096 (2)	0.122 (3)	0.083 (2)	−0.018 (2)	0.0681 (18)	0.0022 (18)
C2	0.0650 (15)	0.0688 (17)	0.0579 (14)	−0.0183 (13)	0.0411 (12)	−0.0044 (12)
C3	0.0470 (13)	0.103 (2)	0.0736 (17)	−0.0075 (14)	0.0351 (12)	−0.0028 (15)
C4	0.0482 (13)	0.089 (2)	0.0539 (13)	−0.0008 (12)	0.0214 (10)	0.0062 (12)
C5	0.0489 (12)	0.0480 (13)	0.0423 (11)	−0.0087 (9)	0.0284 (9)	−0.0037 (9)
C6	0.0566 (12)	0.0370 (12)	0.0496 (12)	−0.0001 (9)	0.0287 (10)	−0.0008 (9)
C7	0.0727 (15)	0.0441 (13)	0.0498 (12)	−0.0088 (11)	0.0330 (11)	0.0017 (9)
C8	0.0499 (11)	0.0375 (11)	0.0446 (10)	0.0009 (9)	0.0289 (9)	−0.0020 (8)
C9	0.0489 (11)	0.0335 (11)	0.0416 (10)	−0.0040 (8)	0.0252 (9)	−0.0030 (8)
C10	0.0509 (11)	0.0345 (11)	0.0411 (10)	−0.0030 (9)	0.0261 (9)	0.0005 (8)
C11	0.0457 (10)	0.0365 (11)	0.0388 (10)	−0.0001 (8)	0.0230 (8)	0.0019 (8)
C12	0.0626 (13)	0.0343 (12)	0.0484 (11)	−0.0039 (10)	0.0324 (10)	−0.0052 (9)
C13	0.0646 (13)	0.0340 (12)	0.0532 (12)	−0.0086 (10)	0.0357 (10)	−0.0012 (9)
C14	0.0434 (10)	0.0454 (13)	0.0403 (10)	−0.0020 (9)	0.0245 (8)	0.0003 (8)
C15	0.0535 (11)	0.0364 (11)	0.0476 (11)	0.0019 (9)	0.0286 (9)	−0.0043 (9)
C16	0.0563 (12)	0.0314 (11)	0.0479 (11)	0.0008 (9)	0.0285 (9)	0.0024 (8)
C17	0.0766 (16)	0.0648 (17)	0.0608 (14)	−0.0097 (13)	0.0422 (12)	0.0107 (12)
C18	0.0734 (16)	0.0681 (17)	0.0530 (13)	0.0017 (13)	0.0397 (11)	−0.0070 (11)

### Geometric parameters (Å, º)

**Table:**

O1—C5	1.380 (2)	C14—C15	1.402 (3)
O1—C8	1.418 (3)	C15—C16	1.378 (3)
O2—C9	1.226 (2)	C1—H1A	0.9600
N1—N2	1.388 (2)	C1—H1B	0.9600
N1—C9	1.342 (3)	C1—H1C	0.9600
N2—C10	1.273 (3)	C3—H3	0.9300
N3—C14	1.372 (3)	C4—H4	0.9300
N3—C17	1.453 (3)	C6—H6	0.9300
N3—C18	1.443 (3)	C7—H7	0.9300
N1—H1	0.8600	C8—H8A	0.9700
C1—C2	1.513 (4)	C8—H8B	0.9700
C2—C7	1.376 (3)	C10—H10	0.9300
C2—C3	1.396 (4)	C12—H12	0.9300
C3—C4	1.375 (4)	C13—H13	0.9300
C4—C5	1.379 (3)	C15—H15	0.9300
C5—C6	1.385 (3)	C16—H16	0.9300
C6—C7	1.392 (3)	C17—H17A	0.9600
C8—C9	1.510 (3)	C17—H17B	0.9600
C10—C11	1.453 (3)	C17—H17C	0.9600
C11—C16	1.398 (3)	C18—H18A	0.9600
C11—C12	1.395 (3)	C18—H18B	0.9600
C12—C13	1.375 (3)	C18—H18C	0.9600
C13—C14	1.405 (3)		
			
C5—O1—C8	117.04 (15)	H1A—C1—H1C	109.00
N2—N1—C9	120.36 (17)	H1B—C1—H1C	109.00
N1—N2—C10	114.47 (17)	C2—C3—H3	119.00
C14—N3—C17	120.51 (19)	C4—C3—H3	119.00
C14—N3—C18	120.91 (19)	C3—C4—H4	120.00
C17—N3—C18	118.58 (19)	C5—C4—H4	120.00
N2—N1—H1	120.00	C5—C6—H6	121.00
C9—N1—H1	120.00	C7—C6—H6	121.00
C3—C2—C7	117.4 (2)	C2—C7—H7	119.00
C1—C2—C3	120.4 (2)	C6—C7—H7	119.00
C1—C2—C7	122.1 (2)	O1—C8—H8A	110.00
C2—C3—C4	121.7 (2)	O1—C8—H8B	110.00
C3—C4—C5	119.6 (2)	C9—C8—H8A	110.00
O1—C5—C6	124.19 (19)	C9—C8—H8B	110.00
O1—C5—C4	115.37 (18)	H8A—C8—H8B	109.00
C4—C5—C6	120.4 (2)	N2—C10—H10	119.00
C5—C6—C7	118.8 (2)	C11—C10—H10	119.00
C2—C7—C6	122.1 (2)	C11—C12—H12	119.00
O1—C8—C9	106.44 (16)	C13—C12—H12	119.00
N1—C9—C8	113.53 (16)	C12—C13—H13	119.00
O2—C9—C8	121.76 (18)	C14—C13—H13	119.00
O2—C9—N1	124.71 (18)	C14—C15—H15	120.00
N2—C10—C11	122.37 (18)	C16—C15—H15	120.00
C12—C11—C16	117.09 (18)	C11—C16—H16	119.00
C10—C11—C12	123.28 (18)	C15—C16—H16	119.00
C10—C11—C16	119.61 (17)	N3—C17—H17A	110.00
C11—C12—C13	121.25 (18)	N3—C17—H17B	109.00
C12—C13—C14	121.74 (18)	N3—C17—H17C	109.00
N3—C14—C15	121.45 (18)	H17A—C17—H17B	109.00
N3—C14—C13	121.50 (18)	H17A—C17—H17C	109.00
C13—C14—C15	117.04 (19)	H17B—C17—H17C	109.00
C14—C15—C16	120.74 (18)	N3—C18—H18A	110.00
C11—C16—C15	122.12 (18)	N3—C18—H18B	109.00
C2—C1—H1A	109.00	N3—C18—H18C	109.00
C2—C1—H1B	110.00	H18A—C18—H18B	109.00
C2—C1—H1C	110.00	H18A—C18—H18C	109.00
H1A—C1—H1B	109.00	H18B—C18—H18C	109.00
			
C8—O1—C5—C4	−174.36 (19)	O1—C5—C6—C7	−178.31 (18)
C8—O1—C5—C6	4.4 (3)	C4—C5—C6—C7	0.3 (3)
C5—O1—C8—C9	169.91 (16)	C5—C6—C7—C2	−0.7 (3)
C9—N1—N2—C10	176.45 (19)	O1—C8—C9—O2	−53.8 (2)
N2—N1—C9—O2	−3.4 (3)	O1—C8—C9—N1	126.83 (18)
N2—N1—C9—C8	175.95 (16)	N2—C10—C11—C12	−10.0 (3)
N1—N2—C10—C11	179.34 (17)	N2—C10—C11—C16	168.4 (2)
C17—N3—C14—C13	−2.1 (3)	C10—C11—C12—C13	178.1 (2)
C17—N3—C14—C15	177.3 (2)	C16—C11—C12—C13	−0.4 (3)
C18—N3—C14—C13	178.3 (2)	C10—C11—C16—C15	−177.2 (2)
C18—N3—C14—C15	−2.3 (3)	C12—C11—C16—C15	1.4 (3)
C1—C2—C3—C4	179.8 (3)	C11—C12—C13—C14	−1.1 (3)
C7—C2—C3—C4	−0.9 (5)	C12—C13—C14—N3	−179.1 (2)
C1—C2—C7—C6	−179.8 (2)	C12—C13—C14—C15	1.5 (3)
C3—C2—C7—C6	0.9 (4)	N3—C14—C15—C16	−179.9 (2)
C2—C3—C4—C5	0.6 (5)	C13—C14—C15—C16	−0.5 (3)
C3—C4—C5—O1	178.5 (2)	C14—C15—C16—C11	−0.9 (3)
C3—C4—C5—C6	−0.3 (4)		

### Hydrogen-bond geometry (Å, º)

Cg1 and Cg2 are the centroids of the C2–C7 and C11–C16 rings, respectively.

**Table:**

*D*—H···*A*	*D*—H	H···*A*	*D*···*A*	*D*—H···*A*
N1—H1···O2^i^	0.86	2.12	2.952 (2)	163
C8—H8*B*···O2^i^	0.97	2.43	3.303 (2)	149
C8—H8*A*···*Cg*2^ii^	0.97	2.65	3.442 (2)	139
C16—H16···*Cg*1^i^	0.93	2.71	3.394 (2)	131

Symmetry codes: (i) −*x*+1, *y*+1/2, −*z*+1/2; (ii) *x*, −*y*+1/2, *z*−1/2.
